# Supplementary material for: Impact of Public Health Education Program on the Novel Coronavirus Outbreak in the United States
Source: Front Public Health. 2021 Mar 15;9:630974. doi: 10.3389/fpubh.2021.630974 (PMC8005517; doi:10.3389/fpubh.2021.630974)
Supplement: Supplementary file 1 [file Data_Sheet_1.PDF]

# Supplementary Material

## 1 PUBLIC HEALTH EDUCATION MODEL

The model is given by the following deterministic system of nonlinear differential equations (where a dot represents differentiation with respect to time $t$):

$$\begin{array}{rcl}
\dot{S}_u &=& -(\lambda_u+\lambda_e)S_u-\psi S_u+\nu S_e,\\
\dot{S}_e &=& -(1-\omega)(\lambda_u+\lambda_e)S_e+\psi S_u-\nu S_e,\\
\dot{E}_u &=& (\lambda_u+\lambda_e)S_u-(\sigma_u+\psi)E_u+\nu E_e,\\
\dot{E}_e &=& (1-\omega)(\lambda_u+\lambda_e)S_e+\psi E_u-(\sigma_e+\nu)E_e,\\
\dot{I}_{us} &=& r\sigma_u E_u+\nu I_{es}-(\alpha_{us}+\gamma_{us}+\psi+\delta_{us})I_{us},\\
\dot{I}_{es} &=& g\sigma_e E_e+\psi I_{us}-(\alpha_{es}+\gamma_{es}+\nu+\delta_{es})I_{es},\\
\dot{A}_u &=& (1-r)\sigma_u E_u+\nu A_e-(\gamma_{ua}+\psi)A_u,\\
\dot{A}_e &=& (1-g)\sigma_e E_e+\psi A_u-(\gamma_{ea}+\nu)A_e,\\
\dot{H}_u &=& \alpha_{us}I_{us}-(\gamma_{hu}+\phi_{hu}+\delta_{hu})H_u,\\
\dot{H}_e &=& \alpha_{es}I_{es}-(\gamma_{he}+\phi_{he}+\delta_{he})H_e,\\
\dot{I}_{cu} &=& \phi_{hu}H_u+\phi_{he}H_e-(\gamma_{cu}+\delta_{cu})I_{cu},\\
\dot{R} &=& \gamma_{ua}A_u+\gamma_{ea}A_e+\gamma_{us}I_{us}+\gamma_{es}I_{es}+\gamma_{hu}H_u+\gamma_{he}H_e+\gamma_{cu}I_{cu},
\end{array} \qquad \text{(S1)}$$

where the forces of infection are given by

$$\lambda_u=\beta\left(\frac{I_{us}+\eta_{A_u}A_u+\eta_{H_u}H_u}{N}\right),\ \ \lambda_e=\beta\left(\frac{I_{es}+\eta_{A_e}A_e+\eta_{H_e}H_e}{N}\right). \qquad \text{(S2)}$$

## 2 BASIC REPRODUCTION NUMBER OF THE MODEL

The model (S1) has a disease-free equilibria (DFE) located within the hyperplane of disease free equilibria, given by

$$\mathcal{D}_0:\ (S_u^*,\,S_e^*,\,E_u^*,\,E_e^*,\,I_{us}^*,\,I_{es}^*,\,A_u^*,\,A_e^*,\,H_u^*,\,H_e^*,\,I_{cu}^*,\,R^*)=(N(0)-(S_e^*+R^*),\,S_e^*,\,0,0,0,0,0,0,0,0,0,R^*),$$

where $N(0)$ is the initial total population size, $S_e^*>0,\ R^*>0$, and $0\le S_e^*+R^*\le N(0)=N^*$. The next generation operator [1, 2] method can be used to analyse the asymptotic stability property of the disease-free equilibria, $\mathcal{D}_0$. In particular, using the notations in [1, 2], it follows that the associated next generation matrices, $F$ and $V$, for the new infection terms and the transition terms, are given, respectively, by

$$F = \begin{bmatrix} 0 & 0 & \beta_u \frac{S_u^*}{N^*} & \beta_e \frac{S_u^*}{N^*} & \beta_u \eta_{A_u} \frac{S_u^*}{N^*} & \beta_e \eta_{A_e} \frac{S_u^*}{N^*} & \beta_u \eta_{H_u} \frac{S_u^*}{N^*} & \beta_e \eta_{H_e} \frac{S_u^*}{N^*} \\ 0 & 0 & Q\beta_u \frac{S_e^*}{N^*} & Q\beta_e \frac{S_e^*}{N^*} & Q\beta_u \eta_{A_u} \frac{S_e^*}{N^*} & Q\beta_e \eta_{A_e} \frac{S_e^*}{N^*} & Q\beta_u \eta_{H_u} \frac{S_e^*}{N^*} & Q\beta_e \eta_{H_e} \frac{S_e^*}{N^*} \\ 0 & 0 & 0 & 0 & 0 & 0 & 0 & 0 \\ 0 & 0 & 0 & 0 & 0 & 0 & 0 & 0 \\ 0 & 0 & 0 & 0 & 0 & 0 & 0 & 0 \\ 0 & 0 & 0 & 0 & 0 & 0 & 0 & 0 \\ 0 & 0 & 0 & 0 & 0 & 0 & 0 & 0 \\ 0 & 0 & 0 & 0 & 0 & 0 & 0 & 0 \end{bmatrix},$$

and,

$$V = \begin{bmatrix} K_1 & -\nu & 0 & 0 & 0 & 0 & 0 & 0 \\ -\psi & K_2 & 0 & 0 & 0 & 0 & 0 & 0 \\ -r\sigma_u & 0 & K_3 & -\nu & 0 & 0 & 0 & 0 \\ 0 & -g\sigma_e & -\psi & K_4 & 0 & 0 & & \\ -(1-r)\sigma_u & 0 & 0 & 0 & K_5 & -\nu & 0 & 0 \\ 0 & -(1-g)\sigma_e & 0 & 0 & -\psi & K_6 & 0 & 0 \\ 0 & 0 & -\alpha_{us} & 0 & 0 & 0 & K_7 & 0 \\ 0 & 0 & 0 & -\alpha_{es} & 0 & 0 & 0 & K_8 \end{bmatrix},$$

where $Q = 1-\omega$, $K_1 = \sigma_u + \psi$, $K_2 = \sigma_e + \nu$, $K_3 = \alpha_{us} + \gamma_{us} + \psi + \delta_{us}$, and $K_4 = \alpha_{es} + \gamma_{es} + \nu + \delta_{es}$, $K_5 = \gamma_{ua} + \psi$, $K_6 = \gamma_{ea} + \nu$, $K_7 = \gamma_{hu} + \phi_{hu} + \delta_{hu}$, $K_8 = \gamma_{he} + \phi_{he} + \delta_{he}$. The control reproduction number is given by

$$\mathcal{R}_c = \rho(FV^{-1}) = \frac{(a_{11} + a_{22})}{2} + \frac{1}{2}\sqrt{(a_{11} - a_{22})^2 + 4\,a_{21}a_{12}}, \tag{S3}$$

where,

$$
\begin{aligned}
a_{11} = &\frac{\beta S_u^* \left(g\nu\,\psi\,\sigma_e + rK_2K_4\sigma_u\right)}{N^*\left(\nu\,\psi - K_3K_4\right)\left(\nu\,\psi - K_2K_1\right)} + \frac{\beta S_u^*\psi\,\left(gK_3\sigma_e + K_2\sigma_u r\right)}{N^*\left(\nu\,\psi - K_3K_4\right)\left(\nu\,\psi - K_2K_1\right)} \\
&+ \frac{\beta\eta_{A_u} S_u^* \left[(1-g)\nu\,\psi\,\sigma_e + (1-r)K_2K_6\sigma_u\right]}{N^*\left(\nu\,\psi - K_2K_1\right)\left(\nu\,\psi - K_6K_5\right)} + \frac{\beta\eta_{A_e} S_u^*\psi\,\left[(1-g)\sigma_e K_5 + (1-r)K_2\sigma_u\right]}{N^*\left(\nu\,\psi - K_2K_1\right)\left(\nu\,\psi - K_6K_5\right)} \\
&+ \frac{\beta\eta_{H_u} S_u^*\alpha_{us}\left(g\nu\,\psi\,\sigma_e + rK_2K_4\sigma_u\right)}{N^*\left(\nu\,\psi - K_3K_4\right)\left(\nu\,\psi - K_2K_1\right)K_7} + \frac{\beta\eta_{H_e} S_u^*\alpha_{es}\psi\,\left(gK_3\sigma_e + K_2\sigma_u r\right)}{N^*\left(\nu\,\psi - K_3K_4\right)\left(\nu\,\psi - K_2K_1\right)K_8} \\
a_{12} = &\frac{\beta S_u^*\left(gK_1\sigma_e + rK_4\sigma_u\right)\nu}{N^*\left(\nu\,\psi - K_3K_4\right)\left(\nu\,\psi - K_2K_1\right)} + \frac{\beta S_u^*\left(gK_1\sigma_e K_3 + \psi\, r\sigma_u\nu\right)}{N^*\left(\nu\,\psi - K_3K_4\right)\left(\nu\,\psi - K_2K_1\right)} \\
&+ \frac{\beta\eta_{A_u} S_u^*\nu\,\left[(1-g)K_1\sigma_e + (1-r)K_6\sigma_u\right]}{N^*\left(\nu\,\psi - K_2K_1\right)\left(\nu\,\psi - K_6K_5\right)} + \frac{\beta\eta_{A_e} S_u^*\left[(1-g)K_1\sigma_e K_5 + (1-r)\psi\nu\sigma_u\right]}{N^*\left(\nu\,\psi - K_2K_1\right)\left(\nu\,\psi - K_6K_5\right)} \\
&+ \frac{\beta\eta_{H_u} S_u^*\alpha_{us}\nu\,\left(gK_1\sigma_e + rK_4\sigma_u\right)}{N^*\left(\nu\,\psi - K_3K_4\right)\left(\nu\,\psi - K_2K_1\right)K_7} + \frac{\beta\eta_{H_e} S_u^*\alpha_{es}\left(gK_1\sigma_e K_3 + \psi\, r\sigma_u\nu\right)}{N^*\left(\nu\,\psi - K_3K_4\right)\left(\nu\,\psi - K_2K_1\right)K_8} \\
a_{21} = &\frac{(1-\omega)\beta S_e^*\left(g\nu\,\psi\,\sigma_e + rK_2K_4\sigma_u\right)}{N^*\left(\nu\,\psi - K_3K_4\right)\left(\nu\,\psi - K_2K_1\right)} + \frac{(1-\omega)\beta S_e^*\psi\,\left(gK_3\sigma_e + K_2\sigma_u r\right)}{N^*\left(\nu\,\psi - K_3K_4\right)\left(\nu\,\psi - K_2K_1\right)} \\
&+ \frac{(1-\omega)\beta\eta_{A_u} S_e^*\left[(1-g)\nu\,\psi\,\sigma_e + (1-r)K_2K_6\sigma_u\right]}{N^*\left(\nu\,\psi - K_2K_1\right)\left(\nu\,\psi - K_6K_5\right)} + \frac{(1-\omega)\beta\eta_{A_e} S_e^*\psi\,\left[(1-g)\sigma_e K_5 + (1-r)K_2\sigma_u\right]}{N^*\left(\nu\,\psi - K_2K_1\right)\left(\nu\,\psi - K_6K_5\right)} \\
&+ \frac{(1-\omega)\beta\eta_{H_u} S_e^*\alpha_{us}\left(g\nu\,\psi\,\sigma_e + rK_2K_4\sigma_u\right)}{N^*\left(\nu\,\psi - K_3K_4\right)\left(\nu\,\psi - K_2K_1\right)K_7} + \frac{(1-\omega)\beta\eta_{H_e} S_e^*\alpha_{es}\psi\,\left(gK_3\sigma_e + K_2\sigma_u r\right)}{N^*\left(\nu\,\psi - K_3K_4\right)\left(\nu\,\psi - K_2K_1\right)K_8} \\
a_{22} = &\frac{(1-\omega)\beta S_e^*\left(gK_1\sigma_e + rK_4\sigma_u\right)\nu}{N^*\left(\nu\,\psi - K_3K_4\right)\left(\nu\,\psi - K_2K_1\right)} + \frac{(1-\omega)\beta S_e^*\left(gK_1\sigma_e K_3 + \psi\, r\sigma_u\nu\right)}{N^*\left(\nu\,\psi - K_3K_4\right)\left(\nu\,\psi - K_2K_1\right)} \\
&+ \frac{(1-\omega)\beta\eta_{A_u} S_e^*\nu\,\left[(1-g)K_1\sigma_e + (1-r)K_6\sigma_u\right]}{N^*\left(\nu\,\psi - K_2K_1\right)\left(\nu\,\psi - K_6K_5\right)} + \frac{(1-\omega)\beta_e\eta_{A_e} S_e^*\left[(1-g)K_1\sigma_e K_5 + (1-r)\psi\nu\sigma_u\right]}{N^*\left(\nu\,\psi - K_2K_1\right)\left(\nu\,\psi - K_6K_5\right)} \\
&+ \frac{(1-\omega)\beta_u\eta_{H_u} S_e^*\alpha_{us}\nu\,\left(gK_1\sigma_e + rK_4\sigma_u\right)}{N^*\left(\nu\,\psi - K_3K_4\right)\left(\nu\,\psi - K_2K_1\right)K_7} + \frac{(1-\omega)\beta\eta_{H_e} S_e^*\alpha_{es}\left(gK_1\sigma_e K_3 + \psi\, r\sigma_u\nu\right)}{N^*\left(\nu\,\psi - K_3K_4\right)\left(\nu\,\psi - K_2K_1\right)K_8}
\end{aligned} \tag{S4}
$$

# REFERENCES


1. Diekmann O. and Heesterbeek J.A.P. Mathenatical epidemiology of infectious diseases: Model building, analysis and interpretation. Wiley, New York., 2000.

2. Van den Driessche P. and Watmough J. Reproduction number and sub-threshold endemic equilibria for computational models of disease transmission. Mathematical Biosciences (2002), (180):29-48.
